# Annotations

**Published:** 1906-12-08

**Authors:** 


### ANNOTATIONS.

### Tobacco Amblyopia.

Although this disease is one which does not frequently occur in the experience of the general practitioner, it is of importance that he should be prepared to recognise the condition whenever he may encounter it. In an excellent post-graduation lecture on the subject Mr. Percy Dunn points out the importance in eye cases of observing all other signs and symptoms of disease in the patient. In cases of this disease the general symptoms are quite typical. The man complains of failing vision, and it will be noted generally that the face is pallid, the hands tremulous, and the tongue furred. He has no appetite for his breakfast, and he will admit to smoking a quarter to half an ounce of shag daily. Distant and near vision are much reduced and are not improved with a lens, and the tests for colour vision reveal a central scotoma for green and red. The ophthalmoscope does not show anything abnormal in the appearance of the discs. The disease usually sets in at middle age, which Mr. Dunn explains not by any increase in the amount of tobacco consumed, but by a defective elimination of the poison resulting from lowered vitality. Treatment resolves itself into the absolute prohibition of every form of tobacco habit and the adoption of means to stimulate the gastric digestion and cure the chronic gastritis. We are glad to find that Mr. Dunn attaches no value to various specific remedies advocated from time to time, and does not even give them a passing reference. The prognosis is good, provided total abstinence from smoking is maintained for at least twelve months. Tobacco chewing may, of course, also give rise to the disease.

### Butter, Genuine and Imitation.

The Committee appointed by Parliament to investigate the manufacture of butter has evidently found that things leave a good deal to be desired. Hence they make several recommendations calculated to secure that the purchaser shall at least not be deceived as to what he is purchasing. One recommendation is that no fat other than butter fat, no vegetable or other oil, and no substance capable of being used as an adulterant of butter shall even be brought into a butter factory; and no fat not derived from milk shall be added to butter at any part of the process of making. This is to prevent the sale of those blended butters—blend being a polite name for adulteration—which are now called simply "butter." So far do the precautions recommended by the Committee go, that they would forbid any fancy name or description which refers to butter or to the dairy industry being affixed to margarine. Probably this would exclude also those delightful pictures of milkmaids and country life by which some brands of fat that never knew a cow are commended to an uncritical public. Margarine is not to be got rid of, for no one disputes its value, but strict precautions are to be taken that it is not sold under a prettier name. When sold by retail it is to be handed to the purchaser in a wrapper on which the word "Margarine" is printed "in black solid capital letters, not less than half an inch square." If the retailer wants a fancy wrapper it must be given in addition to this plain one, and the word "Margarine" must be inscribed on the outer wrapper in as large type as that in which any fancy name he may affix to it is printed. The amount of water permitted in margarine is fixed at 16 per cent., though substances other than butter which contain some portion of butter fat may contain as much as 20 per cent. The Committee's recommendations do not seem to touch the quality of butter itself so long as it is unadulterated, though this is a point of some importance. If honestly carried out, however, they will prevent anything but pure butter being sold under that name.

### The General Medical Council.

In the President's address delivered at the opening of the recent Session of the General Medical Council were two highly satisfactory announcements. The first of these was the intimation that the petition of the British Optical Association to the Privy Council for the grant of a charter, has been refused. The second, that the Sight-testing Opticians' Bill introduced in the House of Lords, and having objects similar to those prayed for by the British Optical Association, has been withdrawn. These decisions are, no doubt, of interest to members of the medical profession, but their chief relationship is to the public. Under existing conditions it is quite open to any spectacle-maker to call himself an optician or any other title which may be held as descriptive of his calling, and similarly it is open to any member of the public who wishes to do so to put the care of his eyesight into the charge of a spectacle-maker. Public opinion in this country approves such an arrangement, and this being so, there is no special need for interference on the part of the medical profession. But when it is proposed to allow a particular group of spectacle-makers, who have passed certain examinations concerned with the preparation and properties of lenses, to pretend to have a legal qualification to distinguish between diseases of the eye and refractive errors, the public interest becomes seriously engaged, and it is high time that those who know the risks which such a practice would involve should interfere. The new proposals involved new dangers, and it is satisfactory to know that in reference to them the Privy Council has listened to the advice of the General Medical Council. A similar result has followed the expression of the Council's opinion in regard to a petition for a charter presented by the "National Association of Medical Herablists"—a proposal even more absurd than that of the spectacle-makers. In advising against these petitions, the Council, of course, has been discharging its function as a guardian of the public interest. It seems necessary frequently to state that the Council does not exist to promote the special welfare of the medical profession. Its action is closely defined by statutory powers, and beyond this it cannot move. In the instances above mentioned it is right that the work and value of the Council should be recognised.

